# Exploring patients’ reasons for participation in a medical education home visit program: a qualitative study in Malaysia

**DOI:** 10.1007/s40037-017-0353-1

**Published:** 2017-04-06

**Authors:** Chai-Eng Tan, Aida Jaffar, Noorlaili Tohit, Zuhra Hamzah, Syahnaz Mohd Hashim

**Affiliations:** 1 Department of Family Medicine, Faculty of Medicine, Universiti Kebangsaan Malaysia Medical Centre, Kuala Lumpur, Malaysia; 2 Department of Primary Care, Faculty of Medicine and Defence Health, National Defence University of Malaysia, Kuala Lumpur, Malaysia

**Keywords:** Home visits, Medical education, Undergraduate, Qualitative, Malaysia

## Abstract

**Introduction:**

Direct contact with patients for medical education is essential in healthcare professional training. Patients who were recruited for a medical education home visit program in Malaysia did so on a voluntary basis without remuneration. This paper aims to explore their reasons for participation in this program.

**Methods:**

An exploratory qualitative study was conducted on patients who had been visited during the 2012/2013 academic session. Purposive sampling was done to select adult participants from varying ethnicities and ages from the list of patients. In-depth interviews were conducted at the participants’ homes and were audio recorded. The transcripts of these interviews were analyzed using thematic analysis.

**Results:**

A total of nine in-depth interviews were conducted. Four main themes were identified from thematic analysis: 1) Perceived meaning of the visit; 2) Perceived benefits and risks; 3) Past healthcare experiences; 4) Availability for visits. The home visits meant different things to different participants, including a teaching-learning encounter, a social visit, a charitable deed or a healthcare check-up. The benefits and risks of accepting unknown students to their homes and sharing their health issues with them had been weighed prior to participation. Prior experience with healthcare services such as gratitude to healthcare providers or having a relative in the healthcare profession increased their receptivity for involvement. Lastly, enabling factors such as availability of time would determine their acceptance for home visits.

**Discussion:**

Patients agree to participate in medical education activities on a voluntary basis for various reasons. Providing good healthcare service and sufficient preparation are crucial to increase patient receptivity for such activities.

## What this paper adds

Patient participation in medical education has been studied in the clinic and ward settings. However, as more programs utilize home visits to teach students, there is a need to explore reasons that increase patient receptivity to educational home visits by healthcare students. This paper provides insights into reasons influencing patient receptivity and suggests pointers for program coordinators to facilitate recruitment of patients for educational home visits.

## Introduction

Home visits provide healthcare students with additional insight into how their illnesses affect their lives, families and communities [1–3]. There are many healthcare issues that are not apparent while the patient is still in the ward or clinic [4, 5]. Various undergraduate and postgraduate programs for health professions have benefited from home visits [4, 6–8]. It has been found that students developed a better understanding of chronic diseases, empathy for their patients, awareness of the inter-relatedness of health and psychosocial issues [4, 9].

The Comprehensive Healthcare module, offered by the National University of Malaysia for second year medical students and third year pharmacy students, utilizes a series of home visits to introduce students to the concept of comprehensive assessment and management of healthcare, particularly for patients with chronic problems living in the community [10]. Unfortunately, organizing home visits for healthcare student educational programs requires considerable effort in identifying suitable patients, logistic arrangements and preparation of the patients, as well as students prior to the visit.

Recruiting patients to accept visits from students, who were practically strangers, was challenging for the module coordinators. In view of the large cohort of students involved annually, the module coordinators needed to recruit a large number of patients each year. These patients were not paid for their time spent on the program. Furthermore, simulated patients were not feasible alternatives for such home visit programs. It is, therefore, interesting to see what influences patients to participate in such home visit programs.

Patient participation in healthcare training is influenced by various factors such as altruism, the desire to contribute, feeling valued, and an opportunity for learning more about their own health [6, 11–13]. However, the home setting involves more risks, by allowing unknown students to enter their homes and loss of privacy.

This study aimed to explore the reasons for patients to agree to participate in home visits within the Comprehensive Healthcare module in an urban Malaysian medical school.

## Methods

This was a qualitative study conducted among adult patients who accepted student visits during the 2012–2013 academic sessions. Only 32 of the total of 48 patients were eligible to be selected for the study on the basis of age more than 18 years, and not having significant cognitive impairment or aphasia. Children who were part of this home visit program also largely consisted of patients with pervasive developmental disorders or mental disability. Therefore, they were not included in this study.

A total of nine in-depth interviews were conducted whereby patients were purposively sampled to obtain a wide variation in terms of age, ethnicity and gender. The semi-structured interviews were conducted based on a topic guide that had been designed to explore patients’ experiences, concerns and expectations from their participation. These interviews were conducted at the patients’ homes and were audiotaped with the patients’ consent. In five of the interviews, the patients’ primary caregivers were also present and contributed their views as well, although the bulk of the data was given by the patients themselves. The caregivers’ accounts were also included for analysis because they substantiated the richness of data from the patients. The audio recordings were then transcribed verbatim for analysis. Interviews conducted in the Malay language were transcribed directly into Malay whereas interviews conducted in Cantonese dialect were transcribed into English to allow analysis by all the researchers. The translations were done by native speakers of Malaysian Cantonese who were also well-versed in English.

NVIVO 10 (QSR) was used to facilitate qualitative data analysis. Coding was done by a single researcher. The transcript was read and coded freely to uncover the reasons for participating in the home visit program. The team members met regularly after every two to three interviews to discuss the codes and the verbatim quotes for the codes. The researchers who conducted the interviews were consulted to verify the accuracy of coding as they were familiar with the context of the interview. Any discordance between the coder and the interviewer was resolved using group consensus. Thematic analyses of the codes were done using an interpretive approach, without using any specific underpinning theory. Diagrams and charting were done to illustrate and consolidate the themes obtained from analysis. Constant comparison was done at each session of analysis to determine whether any new themes were present. The themes were then discussed with a qualitative research expert who was not involved in the project, for peer checking.

As the themes evolved, the study was considered to have attained data saturation when no more new themes emerged from the interviews. This was achieved by the 7th interview and confirmed by two more in-depth interviews. Saturation was achieved despite the small sample size as every interview contained rich data and the objective of the study was specifically to explore the reasons for agreeing to participate in the program. Furthermore, the participants were from diverse backgrounds, providing a variety of views and cultural elements to the findings.

Ethical approval for this study was obtained from the Medical Research Ethics Committee of Universiti Kebangsaan Malaysia Medical Centre.

## Results

A total of nine in-depth interviews were conducted. Table 1 shows the sociodemographic characteristics of the patients who were interviewed. Participants of this study were mainly from an older age group, except for one young man. They were also from varying Asian ethnicities and educational levels which contributed to their different cultural backgrounds and perspectives.

**Table 1:** Participants’ characteristics

Participant	Age (years)	Sex	Ethnicity
P1	61	Female	Malay
P2	65	Female	Malay
P3	71	Male	Chinese
P4	62	Male	Malay
P5	69	Male	Malay
P6	72	Male	Chinese
P7	22	Male	Indian
P8	57	Female	Malay
P9	60	Female	Malay

From analysis, there were four themes regarding the reasons for participation in educational home visits. The themes were ‘perceived meaning of the visit’, ‘perceived benefits and risks’, ‘past healthcare experiences’ and ‘availability for visits’. Table 2 shows selected portions of the transcripts pertaining to each theme.

**Table 2:** Themes

Themes	Transcript
**Perceived meaning of the visit**	
*A contribution to society*	My hope is that they’ll know more and learn more. Firstly is that when you learn more and know more, you are smarter. And you can help other people.– P6 Because, we desire to help. There may be people with similar religions, but each individual’s thinking is different. Not all will say no monetary returns or if they think, ‘I am a patient, and they should consider that if they want information from me, they should give me something. After all, this students are going to graduate and be somebody and earn something more than me, right?’ – P9
*A social visit*	We enjoy it … Even before they come, we are already thinking of how to entertain them. What food should we serve … – P8 In our scriptures it is also mentioned … when visitors come, they bring providence to us. We never know … when they leave, they take away our misfortune … – P1
*A teaching session*	I mean, let them know about my sicknesses and let them study about it. Or if they still don’t understand, they can ask their lecturers … Because I have so many sicknesses, then they will learn more by just interviewing one patient. – P3
**Perceived benefits and risks**	
*Learn more about their health*	It’s best to accept their visit, meaning we get advice from them, become their friends. If they don’t accept their visit, then they don’t get anything. To see a doctor, it’s not easy. – P4
*Emotional benefits*	My father, mother, brother sisters all go to work .. I’m only with two sisters ..that’s why I feel bored .. if they come, I feel better.. someone wants to meet me. – P7 They come to learn, then I get to express my feelings. – P1
*Privacy concerns*	What people say are sensitive questions … For me, I don’t consider certain things as too sensitive. Because to me, it’s better that they know. For example, if they go to a Muslim patient’s home and ask about alcohol consumption, some people may view that as a sensitive issue … For some it’s sensitive, for myself, there’s no need to be sensitive about it. – P9
*Safety concerns*	Well, first of all, you introduced them to come. Then what should I be afraid of? That means if they belong … that if they are your workers or people that you have trained, and they want more experience, there’s nothing to be afraid of. You’re the one who introduced them so why should I be afraid? – P6
**Past experiences with healthcare providers and students**	
*Gratitude*	But we were really grateful because I previously had used up a lot of money before she got treated at U Hospital. – P8
*Connections with healthcare providers or students*	Maybe because God’s giving, my nephew is a doctor. After that, my cousin was a doctor too. – P1
**Availability for visits**	
*Timing*	I can honestly say if they inform me earlier a day or two, I can tell them I would rest on this day and they can come. But the problem comes when my boss accepts a work to renovate a house and he has already decided when to move into the house to work and has given me a job … That means if there’s work to meet the deadline, then I’m not free. If there isn’t any pressure to meet the deadline, then anytime is okay. – P6
*Home setting*	There’s nothing wrong, come to my home, but if I have to go there [hospital], I don’t want to … It’s difficult for me to go there, right? Have to take a taxi, need all that.. – P2 We are seated, relaxed, so they appeared less stressed out (laughs) … At home it’s more relaxed and there’s no rush. – P8

### Perceived meaning of the visit

The students’ home visits were perceived differently by each patient. Participants who viewed the visits as an opportunity to contribute to society did not mind the lack of monetary or other tangible remuneration. However, they believed that payment could be an incentive for other people. Sincerity and satisfaction from helping others were mentioned by the participants. They believed that their participation would lead to the greater good of all as they helped in training future healthcare professionals. Their participation also held meaning and significance to society as well as their students.

Those who perceived the students’ visits as a social visit regarded it as receiving guests to their homes. They were happy to play the role of a host and offer hospitality to the visiting students. Some also held religious views regarding the importance of offering hospitality to guests.

Those who viewed it as a teaching session talked about the importance of students learning from them. They knew that their illness experiences were important to provide practical exposure to the students. They felt that they were experts of their own condition from a patient’s perspective and were willing to be ‘studied’.

Those with previous experiences also recounted how they developed a close relationship with previous students who had visited them. Being remembered by these students supported their perception regarding the significance of their contribution.

### Perceived benefits and risks

The perceived benefits included learning more about their health from the students, and receiving emotional support. The activities that were conducted by the students during the home visit, such as history taking, medication review and counselling, helped them learn more about their health. While expectations from students were low, they valued the opportunity to learn. Receiving visits from such students also proved therapeutic for patients who were lonely. The students were attentive and willing listeners to their problems, offering encouragement to them. This was different from perceiving the visits as a social visit, as these participants were sharing more personal issues with the students. For example, P1 was an old lady with no personal means of transportation, whereas P7 was a paraplegic young man who was unable to go out and socialize like others his age. Both were from the lower income group and physically dependent. They were glad to share their problems with their guests, despite the fact that they were unable to offer much hospitality.

There were potential risks from accepting the visits, such as being open about private or sensitive issues, and personal safety. Participants felt they were able to control the amount of information divulged to the students. Safety was less worrisome because they had been invited by their own healthcare providers and thus had trust in receiving these visits.

### Past experiences

The patients’ past experiences with their personal healthcare provider or even with other healthcare students also influenced their receptivity to the home visits. Some felt gratitude to their healthcare providers and even students. This led them to accept the healthcare students’ visits gladly.

A significant number of participants had relatives in the healthcare sector. Therefore, they would better understand the process of training for healthcare students.

### Availability for visit

When participants have the intention to participate, the final factor of availability becomes a determining factor. This includes suitable timing of the visits so that it will not clash with their own personal schedule. Besides appropriate timing, they need to be in reasonably good health to feel capable of accepting visits from the students.

The home setting is also a determining factor for their availability. For those who find it difficult or a hassle to leave their home, home visits are more acceptable. Some also prefer the atmosphere at home rather than at a healthcare facility, as it is more relaxed and not bound by time constraints.

## Discussion

Some of the reasons for participating in this program echo the findings of previous studies which explored the views of patients who participated in healthcare professional education. The commonly cited reasons were altruism, their role as experts in their illness, as well as weighing the benefits and risks of their participation [12–16]. A few studies also reported that the desire to pay back the healthcare system also played a role in patient involvement in medical education [12, 17]. The current study adds some new insights into the reasons why patients participate in healthcare education activities.

Previous studies have not explored the meaning of accepting educational home visits from healthcare students. A prior phenomenological qualitative study on the meaning of patients’ experiences in medical student teaching was conducted in the clinical setting but not the patients’ own homes [18]. Interestingly, patients perceived the visits as more than the usual teaching-learning encounter when it occurred in the home setting. Patients regarded the students as guests and the encounter as a social visit. This may be tied to the Asian culture, where guests are welcomed and shown hospitality as a token of respect for visitors. For Muslims, their religion also encourages showing hospitality to guests [19]. The participants had already been briefed and informed of the objectives of the module prior to the first visit. Therefore, this perception is not due to misunderstanding of the purpose of the visit. Rather, it is an ingrained aspect of their culture.

Emotional benefits and opportunities to increase their knowledge about their health were valued by the participants, above financial or material remunerations. Similar findings were reported by programs where the home visits were carried out among underserved communities [15, 20, 21]. Concerns about privacy and safety were controlled and thus acceptable to the patients [11]. Hence, selection of patients who would benefit from such visits may improve receptivity to participate in these educational home visits. Educators should also ensure that the students are briefed regarding ground rules for home visits, especially regarding protecting the privacy of the patients and cultural sensitivity.

Some past studies have also mentioned that patients agree to participate in teaching students to ‘repay the system’ [12, 13]. Similarly, the participants of this study described their feelings of gratitude to their doctors or the hospital or even the medical students. While all clinical institutions should provide the best quality of care possible, providing good service may also increase patient receptivity to participate in teaching activities. Most of the patients who were recruited into the program were approached by their own family physicians, with whom they had developed a good doctor-patient relationship. This helped to build trust and willingness to support the teaching activities for healthcare students.

Previous exposure to student teaching encounters also improved their intention to participate in similar activities [22]. The current study also showed similar reasons for participating in such activities. As the patients were receiving healthcare from a teaching institution, they had prior experience of interacting with medical students in the wards or clinics. A few had also received home visits from medical students who were doing their family medicine rotation. This provided them with an understanding and expectation of what will happen during the home visit. Other participants also had relatives who were healthcare professionals or undergoing training in the field. Thus, they understood that clinical exposure with patients is part and parcel of the training required.

Development of a long-term relationship, akin to a family bonding, lasting even after the students graduated was valuable to these patients. This was reported by some of the patients during the in-depth interview. While this did not influence the patients’ willingness to participate in the home visits, it appeared to be an outcome of their participation.

The students would keep in contact and visit once in a while. Being remembered that way made them feel appreciated and respected. Past studies looking into relationships between students and patients have also noted that the students and patients got to know each other personally, aside from the formal educational setting [23, 24]. While there may be concerns regarding ethical boundaries of such relationships, again this could be addressed by laying down ground rules of appropriate boundaries between students and patients [24].

Finally, while physical and time availability is a definite prerequisite for agreeing to participate in the home visit program, some patients actually preferred meeting the students in the home setting rather than at the clinic or hospital. This sentiment was expressed mainly by those patients who had difficulty in accessing healthcare services, such as due to lack of transportation or mobility issues. Another reason for patient preference for the home setting was that the atmosphere at home was more relaxed and less stressful, compared with the busy clinic or ward. Being visited by the students in the home setting reduced the barrier of time constraints as they do not feel rushed to complete the session.

Therefore, what can educators do to improve the acceptance rate of patients for similar educational programs? Selection of patients who enjoyed social interaction or those who were usually isolated due to physical or logistic reasons would possibly offer a two-way benefit to the patients as well as the students. Patients who had prior exposure to healthcare students’ educational activities will probably be more receptive to accepting such invitations. Training institutions for healthcare students also need to ensure that they provide quality patient-friendly clinical services as well. The image of such teaching centres should be emphasized so that patients who seek treatment from them are aware that they may be approached to participate in educational activities. Educators should also be aware of the risks faced by such patients and proactively minimize them by giving appropriate briefing and training to patients and students alike.

This study confirms the findings of some previous studies conducted in other Western countries. Being conducted in a multi-ethnic Asian population, it also shows that culture and spirituality play a role in patients’ decisions to participate in educational home visit programs. This study is limited by its small sample size and the setting of a single teaching institution in Malaysia. Furthermore, participants were among those who had accepted home visits. The opinions of those who had declined such visits were not explored. While the findings may not be generalizable to other settings outside the institution, it may provide valuable insights into ways to facilitate patient recruitment for similar programs. While on-going improvements are being made to the module, further studies to explore barriers of patient participation in such modules are recommended.

## Conclusion

Patient receptivity for involvement in educational home visits are influenced by several factors. When there is no financial reimbursement for their contribution, patient willingness to participate may be influenced by their perceived meaning of the visit, personal consideration of the risks versus benefits, prior experiences with healthcare professionals and students, as well as their availability. Provision of quality healthcare will encourage patient receptivity in educational home visits. Educators should also ensure that adequate briefing and training is given to both patients and students in order to minimize risks to both parties.
